# TG6 Auto-Antibodies in Dermatitis Herpetiformis

**DOI:** 10.3390/nu12092884

**Published:** 2020-09-21

**Authors:** Marios Hadjivassiliou, Timo Reunala, Kaisa Hervonen, Pascale Aeschlimann, Daniel Aeschlimann

**Affiliations:** 1 Academic Department of Neurosciences, Sheffield Teaching Hospitals NHS Trust and University of Sheffield, Sheffield S10 2JF, UK; 2 Department of Dermatology, Tampere University Hospital, 33520 Tampere, Finland; timo.reunala@uta.fi (T.R.); kaisa.Hervonen@uta.fi (K.H.); 3 Celiac Disease Research Center, Tampere University and Faculty of Medicine and Health Technology, 33100 Tampere, Finland; 4 Matrix Biology and Tissue Repair Research Unit, College of Biomedical and Life Sciences, School of Dentistry, Cardiff University, Cardiff CF14 4XY, UK; AeschlimannPC@Cardiff.ac.uk (P.A.); aeschlimanndp@cardiff.ac.uk (D.A.)

**Keywords:** transglutaminase antibodies, TG2, TG3, TG6, dermatitis herpetiformis, gluten ataxia, celiac disease, gluten encephalopathy, gluten neuropathy

## Abstract

Dermatitis herpetiformis (DH) is an extraintestinal manifestation of gluten sensitivity, in which an autoimmune response is directed against transglutaminase 3 (TG3), an epidermal transglutaminase. TG2 is the autoantigen in celiac disease (CD), defined by the presence of enteropathy, and TG6 is the autoantigen in neurological manifestations of gluten sensitivity. The interplay between B cell responses to these 3 transglutaminases in developing the clinical spectrum of disease manifestations is not completely understood. Also, the individual or combined diagnostic and predictive value of the respective autoantibodies is not fully explored. We examined the prevalence of TG6 antibodies in a cohort of patients with DH. TG6 positivity was found in 13/33 (39%), with IgA detected in 11 patients, IgG in 3, and both in 1. This was significantly higher compared to what is seen in the classic CD cases (14%) in a Finnish population. TG6 positive baseline samples constituted 60% of DH patients with no enteropathy (*n* = 10), as opposed to 17% positivity in those with overt enteropathy (*n* = 12; Marsh IIIB). Repeat testing after adherence to a gluten-free diet for 1 year showed reduced titers for TG6 antibodies in 11/13 (85%), whereby 7 patients were now TG6 antibody-negative. Four patients seroconverted and tested positive for TG6 antibodies at one year, due to the ongoing exposure to gluten. We report another patient who presented with neurological manifestations (encephalopathy) leading to the diagnosis of CD, who was intermittently adhering to a gluten-free diet. Serological testing at baseline showed him to be positive for antibodies to all 3 transglutaminases. Eleven years later, he developed DH. He also subsequently developed ataxia and peripheral neuropathy. Although TG3 and TG6 autoantibodies are linked to certain disease manifestations, TG2, TG3, and TG6 autoantibodies can be present across the spectrum of GRD patients and might develop years before onset of symptoms of extraintestinal manifestations. This is consistent with gluten-dependent adaptive immunity being a necessary but not sufficient pretext to organ-specific damage. TG6 antibodies appear to develop more frequently in patients where tolerance to gluten was broken but, either there was no development of the molecular state driving the tissue destruction at the level of the gut, or perhaps more likely, there was more resistance to developing this phenotype.

## 1. Introduction

Gluten-related disorders (GRD) are a group of immune-mediated diseases with diverse manifestations, triggered by the ingestion of gluten [1]. Enteropathy/celiac disease (CD) is not a prerequisite for the diagnosis of GRD, and some patients exclusively present with extraintestinal manifestations in the absence of enteropathy. Such manifestations include skin involvement in the form of dermatitis herpetiformis (DH) and a diverse range of neurological dysfunction, including cerebellar ataxia, sensorimotor axonal neuropathy, sensory ganglionopathy, and encephalopathy characterized by headaches and cognitive difficulties, often with white matter abnormalities on brain imaging [2].

The identification of TG2 as the autoantigen in CD was an important step in our understanding of the pathophysiology of CD [3]. Assessment of serum anti-TG2 antibodies has since become an important tool in CD diagnosis, as a surrogate marker of disease [4]. Recent success in recapitulating the hallmark features of CD including villous atrophy, plasmacytosis, and anti-TG2 autoantibodies in a mouse model shed light on the interplay between gluten, genetics, and IL-15 driven tissue inflammation in the establishment of CD pathology [5]. Importantly, these studies revealed how overexpression of IL-15 leads to activation of intraepithelial cytotoxic T cells, thereby providing a mechanistic explanation regarding the absence of intestinal tissue destruction (‘normal’ gut mucosa), even in the presence of adaptive gluten immunity in some patients. However, despite these advances, the mechanism underlying the clinical spectrum of GRD remains incompletely understood [1]. Variations in the specificity of antibodies produced in individual patients appear to be linked to specific extraintestinal manifestations. The epidermal transglutaminase 3 (TG3) was shown to be the autoantigen in DH [6]. The discovery of another transglutaminase primarily expressed in neural tissue (TG6), that shared enzymatic properties with both TG2 and TG3, offered further insights into the pathophysiology of neurological manifestations of GRD [7]. Patient-derived autoantibodies to these different isozymes are not crossreactive [7,8], and their development appears to be linked to the shared enzymatic properties of these enzymes rather than their structural similarity and potential shared epitopes (for review see [9]). This, therefore, suggests that any of these transglutaminases could be the primary immunological target of the gluten-driven immune response, although relative abundance in the gut and sensitivity to regulation by proinflammatory mediators explains the unique association of TG2 with GRD development.

The potential interplay between B cell responses to these 3 transglutaminases in disease manifestation and individual or combined diagnostic and predictive utility of respective autoantibodies was not fully explored. Here, we examine the prevalence of TG6 antibodies in a well-characterized cohort of patients with DH, and we discuss the implications of the findings for interpretation of patient serology in the wider context of GRD. We also discuss an interesting clinical case that highlights the potential predictive value for these antibodies.

## 2. Methods

### 2.1. Patient Selection

Serology samples from 33 DH patients diagnosed with granular IgA deposits on skin immunofluorescence biopsy were collected at diagnosis, and then 6 months and one year after a gluten free-diet (GFD) at the Tampere University Hospital; 31 patients adhered to the GFD. All patients provided informed consent and the project was approved by the local ethics committee (no specific code was allocated). The project adhered to the ethical principles for medical research, according to the Declaration of Helsinki. Small bowel biopsy was taken at diagnosis and was graded histologically as subtotal or partial villous atrophy with crypt hyperplasia and increased intraepithelial lymphocytes (24 patients), or normal mucosa (9 patients). Sera of a control group consisting of 27 patients with atopic dermatitis and 9 patients with psoriasis were also analyzed.

### 2.2. Serological Testing

Determination of anti-TG6 IgA and IgG was done using our in-house ELISA assays. The methodology was described in detail elsewhere [7,10]. Full-length human TG6 was produced in SF9 cells and diluted to 2 µg/mL in 20 mM Tris/HCl, 300 mM NaCl, pH 7.6, for coating of high-capacity protein binding 96-well plates (Immulon^TM^ 2 HB). All steps to reveal antibody binding were performed according to the published procedure. All serum samples were analyzed in duplicate, on wells containing antigen or only BSA, included on the same plate. The BSA-only background was subtracted from the values for antigen, and the units were calculated from a series of standards run in parallel, whereby a measurement >75 U/mL for IgA or >34 U/mL for IgG was considered to be positive. Standards were calibrated to be consistent with those used in commercial assay produced by Zedira (2nd generation assay). Results are given as the mean of two independent determinations.

## 3. Results

The clinical characteristics of the cohort of patients with DH were already published [11]. In brief, TG3 antibody positivity was seen in 88% (29/33) as compared to 24% (19/79) in patients diagnosed with classic CD. Endomysial antibody (EMA) positivity was found in 79% (26/33) of the DH group. The percentage TG3 antibody positivity dropped from 86% (24/28) to 21% (6/28), after a year on strict gluten-free diet.

**Figure 1:**
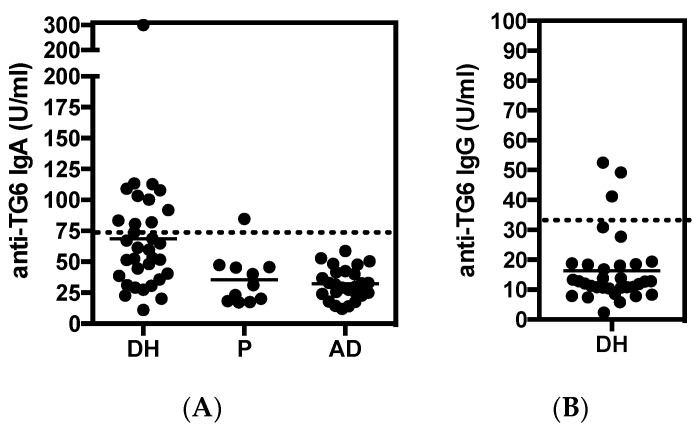
Serum concentration of (**A**) anti-TG6 IgA and (**B**) anti-TG6 IgG in the Dermatitis herpetiformis (DH) cohort (*n* = 33) at baseline. The cut-off limits are indicated by the dashed line. Samples from patients with psoriasis (P; *n* = 9) or atopic dermatitis (AD, *n* = 27) were included as non-Gluten-related disorders (GRD) controls. Note, for one AD sample, no result could be obtained due to unacceptably high non-specific reactivity in the assay.

In the current study, TG6 antibody positivity was found in 13/33 (39%) of DH patients (Figure 1). Eleven were positive for IgA anti-TG6, 3 for IgG, and 1 for both. Sera from patients with unrelated dermatological conditions (36) were also investigated as a control, with one psoriasis patient, testing positive for TG6 IgA (3%). Interestingly, tissue destruction, as determined by the histopathology of intestinal biopsies showed an apparent inverse correlation to serum findings, with TG6 autoantibodies detected in the baseline samples of 6/10 (60%) patients with DH without enteropathy, as opposed to 5/11 (45%) with partial villous atrophy (Marsh IIIA), and 2/12 (17%) in those who had overt enteropathy (Marsh IIIB). Three patients with TG6 antibodies (23%) were serologically negative for anti-TG2 IgA (ELISA & EMA), which is similar to 7/33 (21%) in the cohort as a whole. However, all TG6-positive DH patients also had circulating TG3 IgA.

**Figure 2:**
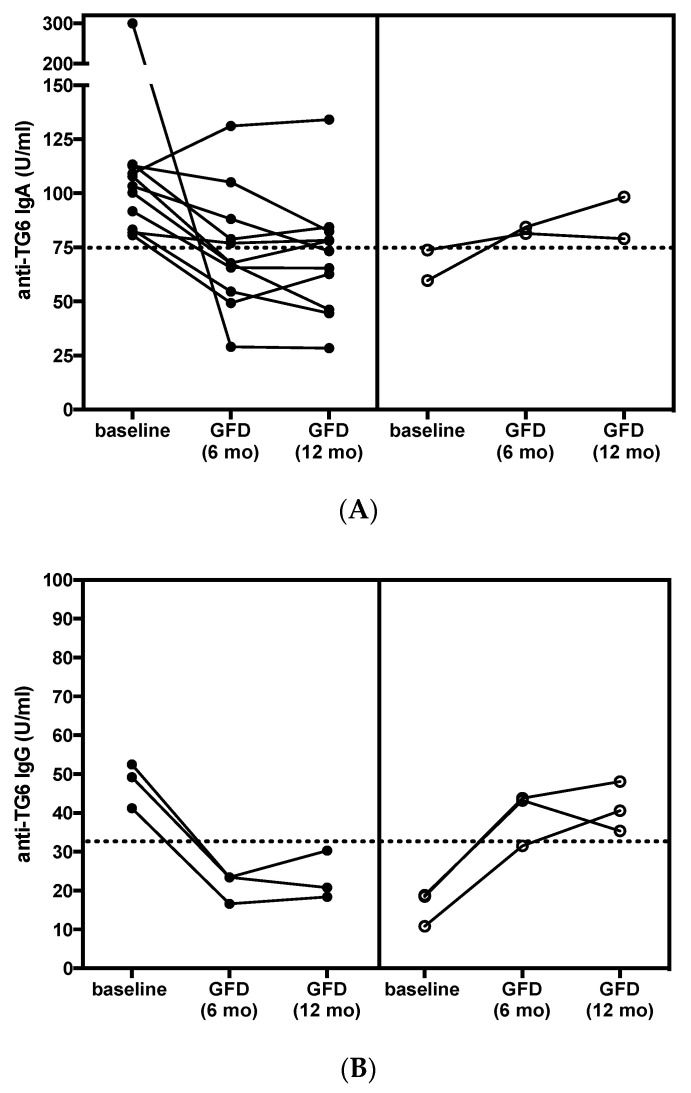
Longitudinal analysis of serum concentration of (**A**) anti-TG6 IgA and (**B**) anti-TG6 IgG in DH patients that tested positive for these antibodies at baseline on a gluten-free diet (GFD) (left panel, closed circles). Patients who tested negative at baseline but subsequently developed antibodies are given in the right panel (open circles). Note—two patients with TG6 IgA failed to respond to GFD, one of which also became positive for TG6 IgG after 6 months of GFD.

Repeat testing after adherence to a gluten-free diet (GFD) for 6 months and 1 year (strict *n* = 28, partial *n* = 3) showed a response in 11/13 (85%) patients (Figure 2). Seven patients who were positive at baseline, tested negative for TG6 antibodies, after 1 year on GFD. One of the two patients that failed to respond to the GFD, also remained positive for anti-TG2 and anti-TG3 IgA, whereas the other patient was only partially compliant with the diet, and although this patient became negative for deamidated gliadin peptide (DGP) antibodies he had persistently high titers of anti-TG3 IgA. Despite this evident effect of gluten withdrawal, and somewhat unexpectedly, the overall TG6 antibody positivity at one year was 8/31 (26%). This was because there were 4 patients who had seroconverted to become positive at one year, 2 for anti-TG6 IgA, and 2 for IgG (Figure 2). This appeared to correlate with ongoing exposure to gluten, as indicated by the other serology (DGP) in two patients (1 IgA and 1 IgG), while no obvious explanation could be found in the other two.

Given the high prevalence of anti-TG6 autoantibodies in this cohort of DH patients, patient records were retrospectively investigated for relevant history. Of the 31 DH patients that were analyzed longitudinally (GFD), six were dead and 25 were followed up to 2011 (mean 21 years), through questionnaire and hospital records. One death from Alzheimer disease was recorded but no other neurological conditions were found in any patient. Autoimmune diseases occurred in four, three had thyroid disease, and one had type 1 diabetes mellitus.

## 4. Predictive Value of Different Transglutaminase Antibodies: An Illustrative Case

**Figure 3:**
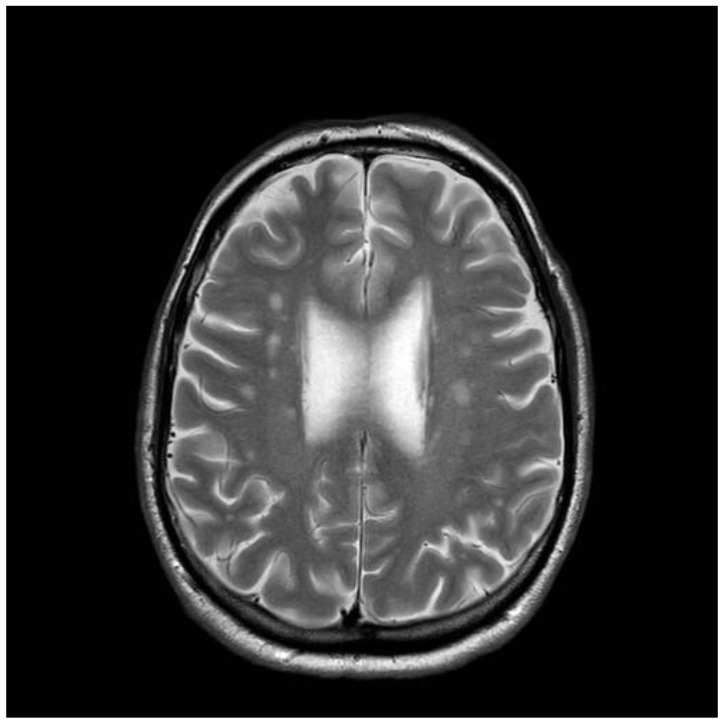
Brain Magnetic resonance imaging (MRI) scan conducted on presentation in 1997. The patient was diagnosed with gluten encephalopathy, having presented with headaches and cognitive difficulties. Serological testing and subsequent duodenal biopsy confirmed celiac disease (CD). The scan shows white matter changes typical of what is seen in the context of gluten encephalopathy.

**Figure 4:**
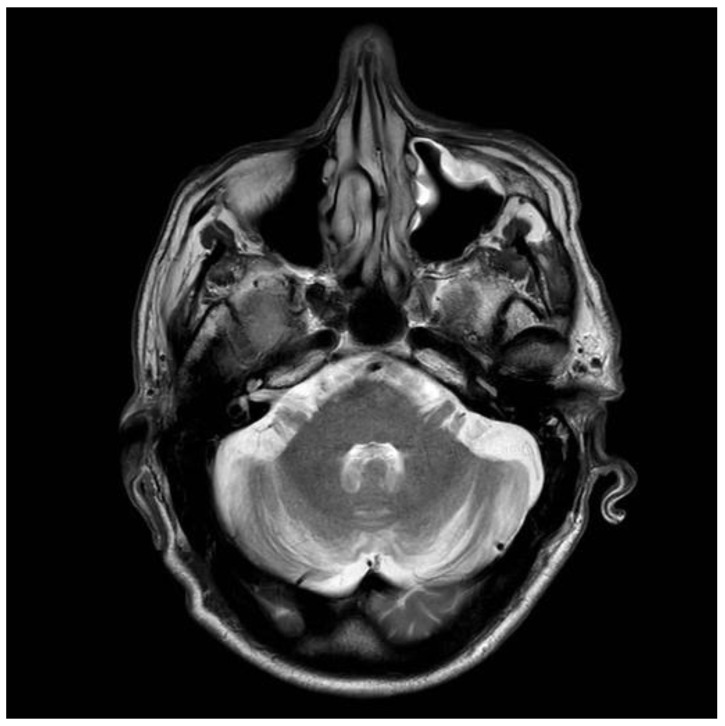
Brain MRI scan conducted on the same patient, as in Figure 3. The scan was conducted in 2018 and at that point showed evidence of cerebellar atrophy that apparently developed over an interval of 21 years. The patient now displays clinical evidence of cerebellar ataxia.

A 41-years old man presented to neurology in 1997, with intractable headaches for the previous 2 years. He described them as severe, often unilateral and throbbing, lasting for several days and often associated with focal but transient neurological deficits (e.g., hemisensory disturbance and double vision). He also complained of memory difficulties and inability to concentrate. Brain MRI was done to rule out any ischemic cerebrovascular events. This showed white matter changes not typical of stroke or inflammation but of undetermined clinical significance (Figure 3). He had no vascular risk factors. He was started on aspirin and discharged. Subsequent outpatient clinical review showed ongoing symptoms of headache, and poor concentration and compromised memory function interfering with his everyday activities. Additional investigations at that stage ruled out systemic lupus erythematosus (SLE) and antiphospholipid syndrome. Cardiac echo and 24-h cardiac recordings, as well as vascular imaging were normal. Blood tests (available at that time) found him to be positive for anti-gliadin (AGA) and EMA antibodies. Duodenal biopsy confirmed the presence of gluten-sensitive enteropathy. It was thought that the headaches and MRI changes were secondary to CD (gluten encephalopathy) [12]. The patient was given advice for GFD by an experienced dietitian. He was followed up at regular intervals (every six months). Initial review after being on the diet for 6 months showed significant improvement in his headaches and cognitive difficulties. His adherence to the GFD over subsequent years was intermittent for a number of reasons—he could not afford gluten-free products, family problems and housing issues, and a one-year spell in prison. During this period, his antibody profile remained positive. He continued to be reviewed by the dietitian and attempts were made to ensure strict adherence to GFD. In 2006, he completely abandoned the GFD, but he restarted it a year later. He gave up the diet again in 2009. A few months later, he presented with an itchy vesicular rash over his arms and face. Dermatological review and skin biopsy confirmed the diagnosis of DH. He was still consuming gluten and serological testing for TG2 IgA, EMA, and anti-gliadin antibodies confirmed the presence of CD-related antibodies. He remained symptomatic with frequent headaches. More recently, he developed a degree of gait incoordination and a tendency to fall. He also complained of distal sensory disturbance with a burning feeling in his feet, less so in his hands. Further brain imaging showed evidence of cerebellar atrophy (Figure 4) that was not present in the baseline scan. Neurophysiological assessment including thermal threshold studies confirmed the presence of small fiber neuropathy.

Serum from this patient was stored at the time of the diagnosis of gluten encephalopathy (1998), and was subsequently available for testing for TG2, TG3, and TG6 autoantibodies, when these serological tests became available [7,13]. The tests showed him to be positive for deamidated gliadin peptide antibodies (IgA/IgG) and, interestingly, all 3 types of transglutaminase autoantibodies (TG2 IgA/IgG, TG6 IgA, and TG3 IgA/IgG). The TG2 antibody positivity would be expected on the basis of CD. TG6 antibody positivity would be in keeping with the diagnosis of gluten encephalopathy and the subsequent development of cerebellar ataxia (gluten ataxia) and neuropathy (gluten neuropathy), due to poor adherence to a GFD. The positive anti-TG3 antibody result from the 1998 sample would explain the subsequent development of DH, which however, manifested in 2009, over 10 years after the initial presentation with the neurological complaints. The patient is still under regular review and during his last attendance (August 2020) he tested positive for TG2, EMA, AGA, and TG6 IgG and IgA antibodies. AGA and TG2 antibodies were tested throughout his clinic appointments and were always positive. An observed reduction of the TG2 titer was only seen in 2008 and 2016, whilst he was trying to be strict with his GFD.

## 5. Discussion

The significance of the serological presence of TG2, TG3, and TG6 antibodies amongst different populations of patients with gluten-related disorders needs clarification. Here, we report a high prevalence of circulating anti-TG6 autoantibodies in DH patients (39%), which was an unexpected finding. It supports the notion that GRD patients, independent of clinical presentation may produce any of these TG isozyme-specific antibodies, or indeed any combination thereof. However, we previously showed that the prevalence of circulating TG6 antibodies in CD patients presenting with ataxia in the UK was much higher than in classic CD patients (73% vs. 40%) [14]. Using the same methodology, the prevalence of TG6 antibodies in Italian pediatric CD patients presenting to gastroenterologists was found to be 25% [15]. Furthermore, in this pediatric cohort of CD patients, a significant correlation between duration of gluten exposure, before the CD diagnosis, and anti-TG6 prevalence/concentration was found [15].

The TG6 antibody prevalence in these 3 groups (patients presenting with neurological manifestations, adult classic CD patients, and pediatric CD patients presenting to the gastroenterologists) is analogous to what was observed in patients with DH. Circulating TG3 antibodies (DH-specific epidermal autoantibodies) were found in up to 87% of patients with DH but in only 24% and 11% of adult and pediatric classic CD patients, respectively [16]. It is noteworthy that in untreated patients with DH, not all patients have circulating anti-TG3 antibodies, yet 100% have IgA-TG3 deposits in the papillary dermis, the site of the primary manifestation [6]. Therefore, although the presence of these antibodies (to TG2, TG3, and TG6) in the serum is diagnostically helpful, their absence does not preclude a localized response at the level of the target tissue (gut, skin, and brain).

Significant advances in understanding of the events leading to autoantibody development were made over the last decade. DH patients were recently shown to have TG3-antibody secreting plasma cells at the level of the gut. Importantly, a gluten challenge of such patients revealed that gluten exposure drove rapid expansion of the TG3-specific plasma cell population in the lamina propria, and their frequency correlated with the serum titer of the corresponding autoantibodies [17]. Furthermore, there was no evidence that this cell population recognizes TG isozymes other than TG3 [17]. This is in keeping with the results from the analysis of patient-derived immunoglobulins, indicating that cross-reacting antibodies are rare [7,8]. Similarly, we analyzed GRD patients presenting with classical CD or gluten ataxia for TG6-specific plasma cells and demonstrated their presence in the lamina propria (Aeschlimann, Dos Reis, Hadjivassiliou, unpublished results). Hence, it is likely that antibodies are generated at the level of the gut; not just TG2 antibodies but also autoantibodies to other TG isozymes. All TG isozymes implicated in GRD form stable thioester complexes with gliadin peptides [18], leading to the uptake and ultimately the presentation of MHC-gliadin complexes by B cells expressing the respective TG-specific IgD. Their activation can consequently be driven through interaction with gluten-specific T cells. In vitro studies confirmed that this was possible [19], and this explanation is consistent with the gut resident T cell response and exquisite gluten dependence of TG autoantibody production, as demonstrated here for TG6 antibodies in DH patients. Enhanced intestinal permeability might drive the immunological reactions that lead to antibody development and circulatory presence [20,21].

How the development of adaptive immunity leads to extraintestinal manifestations remains a matter of debate, although some evidence that the autoantibodies themselves play a role in this has been put forth [9]. Perivascular antibody deposition, as observed in GRD patients can drive organ-specific inflammatory processes that result in tissue damage. TG3 antibodies within immune complexes formed in the papillary dermis of DH patients retain enzymatic activity and thereby drive the innate immune cell activity [22]. Importantly, the demonstration that circulation-derived anti-TG3 antibodies can induce a dermatitis herpetiformis-like pathology in human skin-grafted SCID mice, supports a central role for TG isozyme-specific antibodies in disease establishment in different organ systems [23]. However, expression of anti-TG2 antibodies by themselves did not precipitate CD-like lesions in the small intestine or overt systemic manifestation akin of GRD [24], identifying that establishment of overt tissue damage might require failure in more than one immune regulatory system. The adaptive immune response is a prerequisite for the development of intestinal villous atrophy but is not by itself sufficient. An interplay with the innate immune system that drives IL-15 overexpression in two distinct tissue compartments in the gut is required to mediate tissue destruction [5]. Perhaps this paradigm also applies to the extraintestinal manifestations, with development of anti-TG3 or anti-TG6 antibodies being required for the respective organ-specific manifestations but not being sufficient to precipitate overt tissue damage. This notion is consistent with the observation that the 3 types of autoantibodies could occur across the different forms of GRD, but without apparent clinical correlation. Despite high prevalence of anti-TG6 antibodies in the DH cohort, retrospective analysis of patient history did not reveal any related neurological problems, as was the case in the pediatric CD patients with the anti-TG6 antibodies analyzed previously [15]. We also present a case report that illustrated that the circulating antibodies could indeed be present for long periods of time (>10 years), prior to the onset of the corresponding extraintestinal manifestations. Late onset of DH was also reported in patients that initially presented with classical enteropathy, and appeared to be connected to intermittent GFD (antigen re-stimulation), as was the case here [25,26]. Extraintestinal manifestations were, however, not the consequence of reaching a threshold in the circulating antibody concentration, as there was no correlation between serum concentration and clinical presentation.

Of interest is the potential significance of these antibodies when present in patients who do not seem, clinically at least, to have any obvious corresponding extraintestinal dysfunction. Indeed, our recent work demonstrated that the subgroup of patients with classical CD who are anti-TG6 antibody positive (40% of UK patients) already show a significant reduction in the volume of specific brain regions, when compared to those who are TG6 antibody negative [27]. This finding also suggests that careful investigation (neurological examination and brain imaging) might reveal neurological deficits that are largely ignored, either because they are not considered by gastroenterologists or because the patients would not discuss neurological symptoms in the context of a gastroenterology consultation. Long-term studies of seemingly asymptomatic individuals screened for GRD (e.g., because of family history) who are serologically positive for these transglutaminase autoantibodies, might be helpful in understanding their lifelong significance and help inform the advice given to such patients.

Molecular etiology might also explain the observation that extraintestinal manifestations appear to be a late phenomenon. For example, patients with gluten ataxia and CD presenting with ataxia have a mean age at presentation of 53 years, as opposed to the mean age of 43 years, observed in patients presenting with the classic CD symptoms to gastroenterologists [28]. The prevalence of anti-TG6 antibodies in this Finnish DH cohort was 39%. This figure differed to what was found to be the prevalence of TG6 antibodies in a Finish cohort (same geographical area as the DH patients) of patients presenting with classical CD, which was 12/86 (14%) [14]. Furthermore, we made the observation that TG6 antibody positivity was more prevalent in those DH patients without enteropathy (60%), when compared to those DH patients with overt enteropathy (17%). This is in line with a significant proportion of patients with gluten ataxia and circulating TG6 antibodies displaying none or only minor signs of intestinal tissue damage [14]. TG6 antibodies, therefore, appear to be developed more frequently by patients who lost oral tolerance to gluten but either did not develop the molecular state that leads to tissue destruction at the level of the gut, or perhaps more likely, were more resistant to developing this state due to their genetics.

There were limitations to this study. Ideally, serological testing for all different GRD markers should take place without intermittent sample storage. Longitudinal analysis, as carried out here, is always a compromise between analysis of samples at the point of collection or at a later point when all linked samples for a cohort are available for simultaneous analysis; the latter approach was taken here. In our hands, there is no indication for sample storage (even long-term) affecting measurements of TG6 autoantibodies, as long as the samples were kept in sealed tubes for storage and repeated freeze–thawing cycles were avoided. Such sera are not easy to collect particularly from patients with DH who are becoming increasingly rare. The case described above is a single example of what we observed, and ideally, it should be followed up in a large cohort of patients who do not adhere to a GFD. However, such cases are not common and most patients with CD are likely to adhere to a GFD. Nevertheless, we believe that individual case reports can provide important insights.

## 6. Conclusions

We demonstrated here for the first time that TG6 antibodies are prevalent in patients with DH, and occur at a much higher frequency than what is seen in patients with classical CD. It appears therefore that although linked to certain disease manifestations, TG2, TG3 and TG6 autoantibodies can be present across the spectrum of GRD patients and may develop years before the onset of symptoms of extraintestinal manifestations. Nevertheless, our findings suggest that these antibodies could have a predictive diagnostic value for the future development of specific extraintestinal manifestations. Such autoantibody positivity at diagnosis might further inform the decision to adopt a GFD, particularly for those patients that appear to be asymptomatic but are at risk of developing neurological dysfunction in the longer term.
